# Self-directed digital interventions for the improvement of emotion regulation – acceptability and feasibility for adolescents: systematic review

**DOI:** 10.1192/bjo.2025.10888

**Published:** 2025-11-06

**Authors:** Abigail Thomson, Erin Lawrence, Enxhi Sharxhi, Bonamy Oliver, Ben Wright, Georgina Hosang

**Affiliations:** Centre for Psychiatry and Mental Health, Wolfson Institute of Population Health, Queen Mary University of London, UK; UCL Institute of Education, University College London, UK; East London NHS Foundation Trust, London, UK; City University of London, UK; Queen Mary University of London, UK; NOVA Medical School, Lisbon, Portugal

**Keywords:** Emotion regulation, child and adolescent psychiatry, evidence-based mental health, digital interventions, eHealth

## Abstract

**Background:**

In-person, therapist-supported interventions targeting emotion regulation have been shown to improve the mental health of adolescents. Increasingly, self-directed digital interventions (e.g. mobile apps) are being developed as a cost-effective, scalable solution to widen access to support. However, evidence of the acceptability and feasibility of these interventions has yet to be synthesised.

**Aims:**

To identify existing evidence on the benefits, acceptability and feasibility of self-directed digital interventions that target emotion regulation in adolescents (aged 11–18 years).

**Method:**

A Preferred Reporting Items for Systematic reviews and Meta-Analyses (PRISMA)-guided systematic review was conducted to identify studies published from 1 January 2010 to 13 November 2024 investigating self-directed digital emotion regulation interventions for adolescents. A total of ten electronic databases were searched (e.g. PsycInfo). Data on the effects, and perceived acceptability, of the interventions were extracted, with results narratively synthesised. Methodological quality was assessed using the Effective Public Health Practice Project Quality Assessment tool.

**Results:**

Six out of 9049 studies met the eligibility criteria and included preliminary evidence on self-directed digital interventions that target emotion regulation, in a pooled sample of 1271 adolescents. All interventions identified were brief (most <1 month) and included different components to target emotion regulation (e.g. mindfulness, mood monitoring). Most interventions demonstrated benefits for emotion regulation and were acceptable for use by an adolescent population.

**Conclusions:**

Although the evidence base was small, the included studies demonstrate preliminary evidence of the benefits and acceptability of self-directed, digital interventions for emotion regulation in adolescents. Future research should focus on approaches beyond mindfulness, including components to target the related skills required to access emotion regulation strategies (e.g. emotional awareness) and use them flexibly.

Adolescence is a period of rapid physical, psychological and social development. Approximately 75% of mental illnesses emerge during this period,^1^ with many persisting into adulthood, producing significant long-term consequences for an individual’s social adjustment, physical health, overall functioning (e.g. sleep) and quality of life.^2^ In the UK, 1 in 6 adolescents (aged 11–16 years) have been identified as having a probable mental illness – a figure that has steadily increased in the past decade.^3^ While the exact reasons for this increase are still uncertain, its effects on adolescents and society are a major concern for practitioners, researchers and policy-makers alike.^4^ Current attempts to support this population are largely designed to target specific conditions (e.g. depression^5^). However, 60% of adolescents with one diagnosable mental illness have one or more additional conditions.^6^ Mental health comorbidity – the presence of two or more mental illnesses in an individual – is the rule rather than the exception, and has been associated with greater clinical severity and a poorer overall quality of life.^7^ Transdiagnostic interventions are designed to be directly effective across several mental illnesses, altering psychopathological processes common to multiple conditions (e.g. emotion regulation).^8^ Emerging evidence suggests that this approach is effective in targeting diverse psychopathologies, activating a range of related, beneficial developmental cascades, including improvement in social and academic outcomes.^9^ A transdiagnostic approach to treatment is also considered to be time- and cost-effective compared with disorder-specific strategies, and may offer a more sustainable alternative to treatments currently available to this population.^7^

## Emotion regulation as a transdiagnostic mechanism

**Fig. 1:**
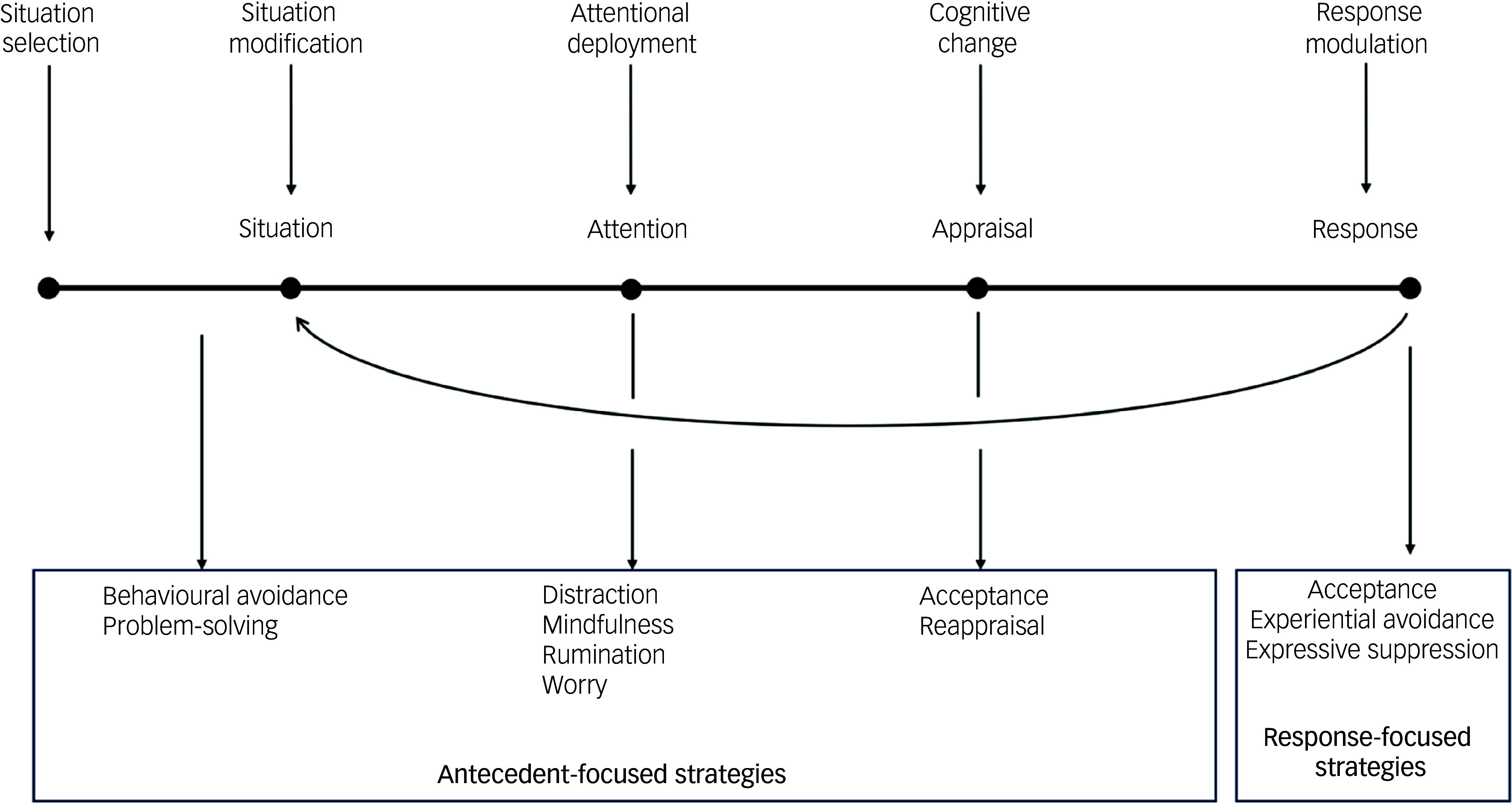
Gross’s Process Model of Emotion Regulation.^12^ Situation selection can be understood as an individual’s efforts to alter the likelihood of being in an emotion-evoking situation. Situation modification involves modifying a situation at the time to change its emotional impact. Attentional deployment involves directing attention towards or away from an emotion or its causes. Cognitive change enables reappraisal of a situation to change its emotional significance. Response modulation includes any efforts to modify the behavioural, experiential and physiological elements of an emotional response.^12^

Emotion regulation has received increased attention in recent years as a transdiagnostic mechanism and clinical target in psychological treatment.^10^ Although the concept of emotion regulation remains unclear by definition, it can be broadly understood as a goal-directed, multidimensional process wherein an individual monitors, evaluates and shapes their emotions when they have them, and how they internally experience or outwardly express them.^10,11^ There have been several different conceptualisations of emotion regulation, but by far the most influential is the Process Model of Emotion Regulation.^12^ According to this model, an individual recognises an emotion regulation goal (e.g. to communicate to others; to modify behaviour), selects and finally implements specific emotion regulation strategies.^13^ Gross defined a set of five distinct emotion regulation processes occurring at different points in an emotional experience (see Fig. 1): situation selection, situation modification, attentional deployment, cognitive change and response modulation.^13^ Each can be understood to influence an individual’s emotional response in a way that can be interpreted as either adaptive (e.g. problem-solving, acceptance) or maladaptive (e.g. withdrawal, suppression^12^), depending on the context.

Maladaptive patterns of emotional experience or expression are typically understood as emotion dysregulation and have physiological, cognitive and social consequences.^12,13^ Evidence demonstrates that emotion dysregulation is present across a range of psychopathologies, including both internalising (e.g. generalised anxiety disorder, major depressive disorder, dysthymia) and externalising disorders (e.g. attention-deficit hyperactivity disorder (ADHD), conduct disorder, oppositional defiant disorder).^14^ Recent findings also indicate a significant shift in emotion regulation between ages 13 and 15 years (e.g. access to strategies, use of adaptive versus maladaptive strategies), suggesting that adolescence is a particularly vulnerable period in the development of emotion regulation.^15^ Therefore, interventions targeting emotion regulation as a transdiagnostic construct central to the development and maintenance of psychopathology may reduce the risk and severity of adolescent psychopathology.

## Emotion regulation as a clinical target

Existing psychological interventions adopt different approaches to improving emotion regulation. Some focus on reducing the use of emotion regulation strategies that may be understood as maladaptive, such as rumination (e.g. rumination-focused cognitive behavioural therapy, RF-CBT),^16^ while others focus on increasing the use of strategies that may be understood as adaptive, such as acceptance (e.g. acceptance and commitment therapy (ACT^17^)). Others move beyond modifying the use of specific strategies and instead focus on developing wider emotion regulation skills (e.g. identifying and labelling emotions, understanding the context in which emotions occur, applying distress tolerance techniques – for example, dialectical behaviour therapy (DBT^18^)). Much of the research to date has focused on the effectiveness of in-person emotion regulation interventions, despite a growing number of self-directed digital solutions (e.g. mobile apps) available for adolescent emotion regulation and psychopathology.^19^ Such interventions are led by the service user, with little to no support from anyone else (e.g. therapist, parent/carer), and aim to widen access to support for adolescents. Some attempts have been made to examine the effectiveness of digital interventions targeting emotion regulation in adolescents, and emerging findings demonstrate that, in general, such interventions (e.g. digital games, virtual reality therapies) are effective in improving emotion regulation.^20^ However, the effectiveness of interventions delivered in this self-directed format is, as yet, unclear. It is thought that this approach has a greater capacity for innovation and engagement with adolescents,^21^ and the potential to extend effective care cost-effectively and sustainably,^22^ but more research is needed to determine how such interventions can be applied at scale to support this population.

## Review objectives

This systematic review investigates evidence on current self-directed digital interventions developed for adolescents (aged 11–18 years), and their effects on emotion (dys-)regulation, psychopathology and functioning (e.g. academic achievement). The review provides an important extension to existing work that has thus far demonstrated the effectiveness of in-person or therapist-supported interventions available for young people (aged 6–24 years),^14,19^ as well as the utility of a broad spectrum of digital emotion regulation interventions for adolescents.^20^ This review takes a more specific focus to develop evidence on a burgeoning number of self-directed, scalable, digital mental health interventions available to adolescents with or without diagnosed psychopathology. This is a timely contribution, employing a systematic review approach to provide a robust insight into the existing evidence base that underpins the growing number of publicly available emotion regulation mobile apps developed to provide mental health support at scale to young people.^23^

Specifically, we sought to answer the following research questions:

- Are current self-directed digital interventions that target emotion regulation acceptable and feasible for use within an adolescent population?
- Do self-directed digital interventions that target emotion regulation in adolescents have benefits for psychopathology and overall functioning (e.g. academic achievement)?
- How do the components of current self-directed digital interventions map onto existing theory and models of emotion regulation?

## Method

This systematic review was registered in PROSPERO, the International Prospective Register of Systematic Reviews (no. CRD42022385547). The primary amendment to this protocol^24^ is the decision not to undertake a meta-analysis due to the methodological and clinical heterogeneity of the included studies. We also decided to include a new database (ACM Digital Library) after the review was registered. No further amendments were made. The review was conducted according to the procedure and requirements described in the Preferred Reporting Items for Systematic Review and Meta-Analysis (PRISMA) guidelines.^25^ The completed PRISMA checklist is provided in Supplementary File 1 available at https://doi.org/10.1192/bjo.2025.10888.

### Search strategy

The search strategy (provided in full in Supplementary File 1) was designed to identify all studies examining one or more self-directed digital mental health interventions for adolescents and that include at least one component to target emotion (dys-)regulation (i.e. strategy or skill). It searched for synonyms for the following three concepts: adolescents, emotion (dys-)regulation and self-directed digital interventions. The strings were combined based on the population (i.e. adolescents aged 11–18 years), intervention format (i.e. self-directed digital interventions) and intervention target (i.e. emotion regulation) of interest. Limitations were placed on the publication date (i.e. from 2010) and language (i.e. English). A body of research addressing this topic was identified before the search through word of mouth, key reference lists and simple searches to check iterations of the strategy. The search was revised until it was sufficiently sensitive to capture the pre-identified studies.

### Information sources

Ten electronic databases were searched for studies published from 1 January 2010 to 13 November 2024: MEDLINE, PsycInfo, Global Health, Scopus, Web of Science Core Collection, EBSCO CINAHL, EBSCO ERIC, Ovid Embase, The Cochrane Central Register of Controlled Trials (CENTRAL) and ACM Digital Library. ‘Grey’ literature, such as preprints and theses, was also included in this review (databases: HMIC, EThOS, PsyArXiv, Trip, ClinicalTrials.gov). This search was updated periodically to identify any new relevant research from the selected databases (most recent search, 13 November 2024); subsequently, no further studies were identified for inclusion.

### Eligibility criteria

Studies were screened according to set eligibility criteria (Table 1). Studies were eligible for inclusion if the intervention included at least one component to target emotion regulation (i.e. an emotion regulation strategy or skill), and if the study included a valid measure of emotion regulation. Studies that did not measure emotion regulation using a validated measure were excluded from this review, to protect against the inclusion of evidence that is not robust. Also excluded were studies that measured a related but distinct construct (e.g. self-regulation, coping), and those that did not provide any evidence on the effects of the intervention on emotion regulation. Studies must have reported primary research with direct contact or observation of individuals, but there were no further restrictions on study design, setting or geographical location.

**Table 1:** Eligibility criteria for screening

Criteria	Inclusion	Exclusion
Population	Any adolescent (aged 11−18 years) With or without a diagnosed mental health problem	Non-human participants
Intervention	Must include at least one component to target emotion (dys-)regulation (i.e. strategy or skill) Any intervention delivered entirely by digital technology (e.g. app based, web based, etc.) Can be delivered in any appropriate setting such as at home, in school, in voluntary and community settings, primary, secondary or tertiary care Must be self-directed	Emotion (dys-)regulation is not measured The intervention is not entirely self-directed (i.e. it includes modules or components that are not completed by the adolescent or are completed with support from a clinician, teacher, parent or caregiver)
Comparison	Employing a control group is not an essential criterion for inclusion in the review.	
Outcomes	Must measure emotion (dys-)regulation as an outcome using a validated measure	
Study	Publication dates from 1 January 2010 to 3 March 2024 Studies from any geographical location Primary research study with direct contact or observation of individuals ‘Grey’ literature, theses, reports, preprints	Non-English language Study protocols or descriptive studies such as case reports/studies that do not include direct contact or observation of individuals Review papers, books, editorials, commentaries, poster abstracts, conferences

### Study selection

All identified studies were exported to Endnote 20.5 for macOS (Clarivate, London, UK; https://endnote.com/downloads/), and any duplicates were removed following a specified method^26^ before being imported into Rayyan^27^ for further de-duplication and screening. Abstracts and titles of identified studies were screened by A.T. as a primary screener based on the eligibility criteria. A total of 50% of articles were jointly assigned for second screening at the title and abstract stage (E.L. screened 10%, E.S. screened 15%, J.L. screened 25%). There was strong agreement between screener pairs (E.L. + A.T., *κ* = 0.99; E.S. + A.T., *κ* = 0.99; J.L. + A.T., *κ* = 0.98). Disagreements (*n* = 35) regarding the inclusion of a study were discussed, and research articles reviewed, until a consensus was reached. Those studies that met the eligibility criteria entered the full-text screening stage for further checks against the eligibility criteria. Full-text screening allowed for the identification of those interventions that did not measure emotion (dys-)regulation directly. All articles were independently screened by A.T. and E.S. at the full-text stage. There was strong agreement between screener pairs (*κ* = 0.86). Disagreements (*n* = 1) regarding the inclusion of a study were discussed, and research articles reviewed, until a consensus was reached.

### Data extraction and management

Data were extracted and collated from eligible studies by two independent reviewers (A.T. and E.L.), and tracked in Microsoft Excel using a structured coding form and associated coding manual. Information relating to study characteristics (e.g. author(s), publication date), participant characteristics (e.g. age, gender), digital intervention characteristics (e.g. name, focus) and relevant clinical and emotion dysregulation outcomes were extracted from each study. Data were first extracted on 16 August 2023. Study investigators were contacted for missing/unreported data or additional details, as required.

### Outcomes

The primary outcome of this review is the change in emotion (dys-)regulation occurring as a result of participation in a self-directed digital intervention that addresses emotion (dys-)regulation. Emotion (dys-)regulation must be assessed and, where possible, using a valid and appropriate item, scale or measure (e.g. Child Social Behaviour Questionnaire),^28^ including through clinical interviews or self-reported measures. An existing review of emotion (dys-)regulation assessment^29^ and similar reviews^30,31^ were used as guidance to determine a measure’s eligibility. The measure must have been cited as valid by at least one of these reviews to be adjudged eligible. Effect sizes were extracted for measures of emotion regulation (or calculated when data were available) and interpreted according to Cohen’s conventions.^32^ Other outcomes of interest in this review were the change in psychopathology and functioning (e.g. academic achievement). Symptoms of psychopathology were assessed by any available valid and appropriate measure, including through clinical interview or self-reported measures. Where data were available, information about the acceptability and feasibility of the interventions was also collated.

### Quality and risk of bias assessment

Information to determine any study bias was also collated. Two researchers (A.T. and E.L.) independently assessed the methodological quality of the included studies using the Effective Public Health Practice Project quality assessment tool (EPHPP). EPHPP is applicable to a range of quantitative study designs (e.g. case–control studies), and has been judged particularly suitable for systematic reviews on the effects of interventions/treatments.^33^ Evidence has shown that EPHPP has good content and construct validity.^33,34^

### Data synthesis

Meta-analyses could not be undertaken due to the heterogeneity of interventions, study designs and outcome measures. There were also too few studies for synthesis into comparable groups. As such, a narrative synthesis of the results was conducted, guided by recommendations from Popay and colleagues.^35^ The effects of identified interventions are summarised based on the specific components employed to target emotion regulation, drawing attention to notable differences in the conceptualisations of emotion regulation as a clinical target (i.e. emotion regulation strategies, emotion regulation skills and deficits). Specific attention is also given to the acceptability and feasibility of the self-directed digital intervention format. Data from these studies, including significance and direction of effects, are presented in summary tables. Given the range of outcome measures and statistical approaches applied across studies, results should not be compared across interventions.

## Results

**Fig. 2:**
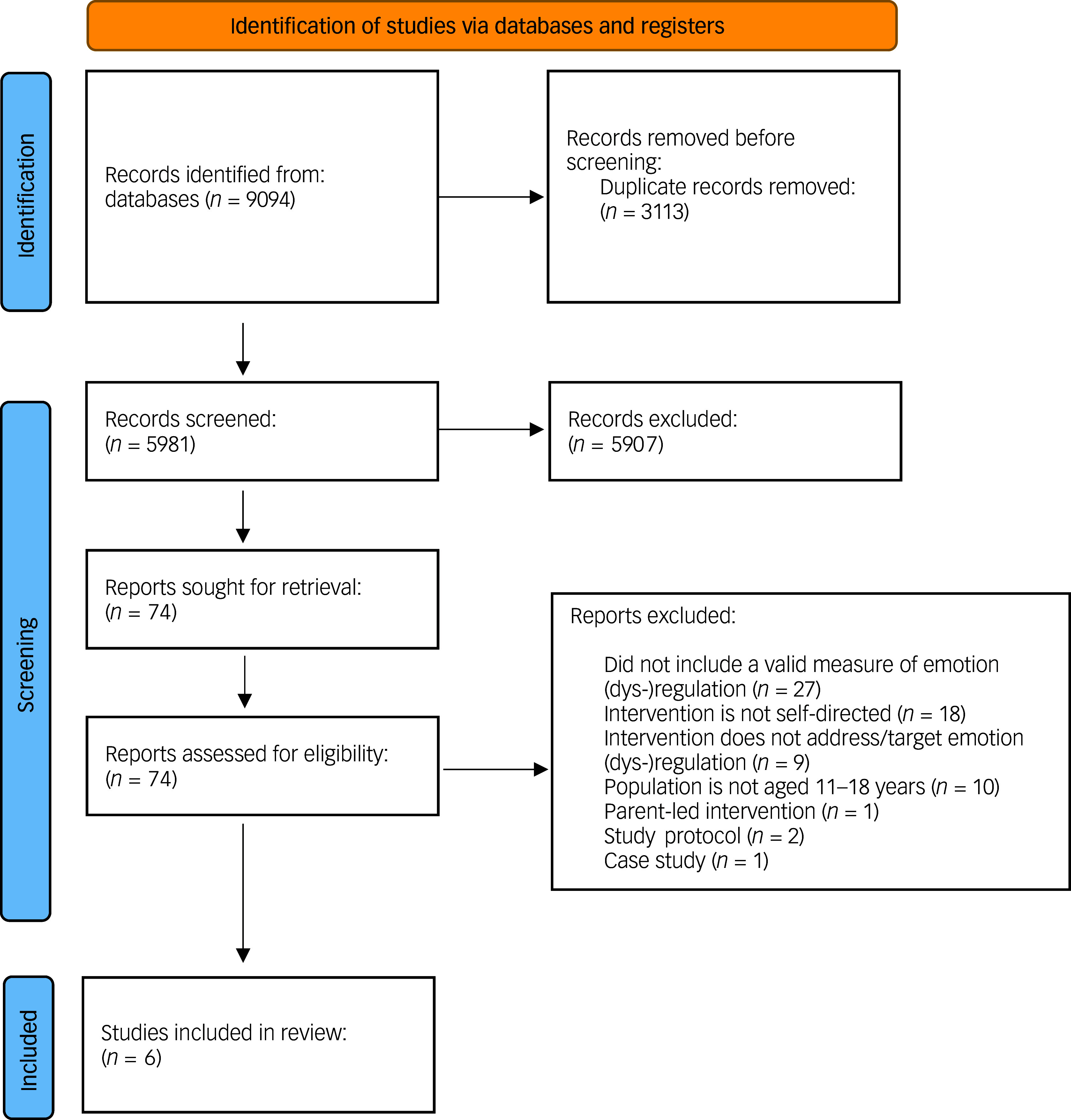
Study selection.

A total of 9094 records were retrieved from the database searches (see Fig. 2 for the study selection process). Following the removal of 3113 duplicate records, the abstracts and titles of the remaining records were screened according to the eligibility criteria. At the abstract and title screening stage, 5907 records were excluded. In total, 74 papers entered the full-text screening stage, of which 6 met the eligibility criteria and provided sufficient data.^36–41^

### Characteristics of included studies

The characteristics of the included studies are summarised in Table 2. Most (*k* = 5) were conducted in the USA and employed a sample of between 80 and 618 adolescents aged 12–20 years. Studies were a mixture of brief cohort (one group pre- + post-test) studies (*k* = 3^36,39,40^) and randomised controlled studies (RCTs) (*k* = 3^37,38,41^), comparing the intervention with either an active or wait-list control. Two studies tested the same intervention – the first was a cohort study to test preliminary feasibility and acceptability^36^ and the second was a pilot RCT to confirm the intervention’s effects.^37^ Across most studies (*k* = 5), investigating the effects of a self-directed digital intervention in improving emotion regulation was the focus. However, for one study, although the primary focus was on mental health symptoms (depression and anxiety), emotion regulation was captured as a secondary outcome.^41^ A range of measures were used to capture change in emotion regulation as a result of using the intervention (Table 2). Only half of the studies^36,37,41^ investigated the impact of the intervention on improving psychopathology, but most measured at least one related functional outcome (i.e. stress, worry, life satisfaction, self-control), although the types of outcomes measured differed across studies.^36,38–41^ Results from the assessment of risk of bias are shown in Table 2. The quality of most of the included studies was weak or moderate. The uncontrolled study design was one of the primary reasons for the lower quality ratings, as well as the lack of blinding and missing information about confounders in some studies. Full ratings for each included study are included in Supplementary File 2.

**Table 2:** Study characteristics

Study details: authors, year, country	Study aims	Study design	*N*	Age, years (mean)	Ethnicity	Gender	Emotion regulation measure	Psychopathology measure	Quality
Hilt and Swords (2021) USA^36^	To determine both the acceptability and initial effects of a brief intervention (mobile app) for ruminative adolescents, to reduce both rumination and internalising symptoms	Cohort (one group pre + post)	80	14.1	86.3% White, 2.5% Native American, 1.3% Black, 1.3% multiracial	53.8% male, 45% female, 1.2% prefer not to say	CRSQ	CDI, MASC, PSC^a^	Weak
Hilt et al (2023) USA ^37^	To determine whether a brief app-based intervention, which involves both mood monitoring and mindfulness, would uniquely reduce rumination, depression and anxiety, compared with a mood-monitoring-only version of the app	Pilot RCT, active control	152: 72 active, 80 control	13.7	82.2% White, 10.5% multiracial 3.3% Black or African American, 2.0% Asian or Asian American, 1.3% other, 0.7% Native Hawaiian or Pacific Islander	41.5% male, 58.5% female	CRSQ	CDI, MASC, PSC^a^	Moderate
Houck et al (2022) USA^38^	To examine whether adolescents who completed the tablet-based iTRAC intervention, relative to a wait-list comparison, demonstrated differences in emotional awareness, intentions to use emotion regulation strategies, the use of emotion regulation strategies and other indicators implicated in emotion regulation processes, such as beliefs about emotions and emotional self-efficacy	Pilot RCT, wait-list control	85: 42 active, 43 control	12.7	40% Black, 33% White, 10% multiracial, 4% other categories, 14% did not respond	44% male, 56% female	ERBS-R DERS ADS ASRI-P^a^ ERC^a^	Not measured	Strong
Kuosmanen et al (2017) Ireland^41^	To explore the feasibility of delivering a computerised cognitive behavioural therapy gaming intervention (SPARX-R) for young people in an alternative education programme in Ireland	Cluster RCT, wait-list control	146: 92 intervention, 54 control	17.6	Not measured	48.9% male, 51.1% female	ERQ	SMFQ, GAD-7	Weak
Mrazek et al (2019) USA^39^	To explore whether a digital mindfulness course, designed specifically for implementation in high schools, had beneficial outcomes for students and could be feasibly delivered in a school setting	Cohort (one group pre + post)	190	15.1	49.5% Asian or Asian American, 37.4% Caucasian, 8.9% multiracial, 1.6% Black or African American, 1.1% American Indian/Alaskan Native, 1.1% prefer not to say, 0.5% Native Hawaiian or Pacific Islander	55.8% male, 41.1% female, 1.6% prefer not to say, 0.5% non-binary	ERQ-CA	Not measured	Weak
Schnitker et al (2021) USA^40^	To examine the effectiveness of the CharacterMe intervention across a 6-month period.	Cohort (one group pre + post)	618	16.1	41.1% Asian or Asian American, 29.5% Latino, 12.9% White, 11.5% other, 5.0% African American	43.4% male, 56.6% female	CEMS	Not measured	Moderate

ADS, Affect Dysregulation Scale; ASRI-P, Adolescent Self-Regulatory Inventory–Parent report; CDI, Children’s Depression Inventory; CEMS, Children’s Emotion Management Scales; CRSQ, Children’s Response Styles Questionnaire; DERS, Difficulties in Emotion Regulation Scale; ERBS-R, Emotion Regulation Behaviours Scale – Revised; ERC, Emotion Regulation Checklist; ERQ, Emotion Regulation Questionnaire; ERQ-CA, Emotion Regulation Questionnaire for Children and Adolescents; GAD-7, Generalised Anxiety Disorder Rating Scale; MASC, Multidimensional Anxiety Scale for Children; PSC, Paediatric Symptom Checklist; RCT, randomised controlled trial; SMFQ, Short Mood and Feeling Questionnaire.

a. Parent report.

### Intervention characteristics

The characteristics of the included interventions are summarised in Table 3. All interventions were brief and were in the early stages of development and testing. The active intervention period in all studies lasted between 14 and 49 days, although some studies allowed participants to engage with the intervention beyond this point.^36,37,41^ Some interventions offered shorter daily activities (1–15 min^36,37,40^), while others comprised longer modules (20–45 min each) that participants completed once per week.^38,39,41^

**Table 3:** Intervention characteristics

Study details: authors, year	Name	Focus	Device	Setting	Duration of active intervention use	Duration of activities	Emotion regulation component	Monitoring adherence
Hilt and Swords (2021)^36^	CARE app	To reduce repetitive negative thinking (rumination) and internalising symptoms	Mobile app	Wherever the participant chooses	21 days^a^	Participants advised to engage three times per day; activities are between 0 and 15 min	MBSR: mindfulness activities (e.g. breathing, body scan), mood monitoring	Monitored electronically through in-app technologies; push notifications; small financial bonus offered to those who used the app as per protocol
Hilt et al (2023)^37^	CARE app	To reduce repetitive negative thinking (rumination) and internalising symptoms	Mobile app	Wherever the participant chooses	21 days^a^	Participants advised to engage three times per day; activities are between 0 and 15 min	MBSR: mindfulness activities (e.g. breathing, body scan), mood monitoring	Monitored electronically through in-app technologies; push notifications; small financial bonus offered to those who used the app as per protocol
Houck et al (2022)^38^	Internet-based iTRAC	To enhance emotion regulation skills in reducing poor decision-making that can lead to unplanned sex or experimentation with drugs and alcohol	Tablet	A designated classroom at school	28 days	4 modules, each of 30–45 min	Learning abilities related to emotion regulation (e.g. emotional awareness, recognising somatic cues); learning and applying three strategies for emotion regulation: ‘get out’ (getting away, physically or cognitively, from triggers for strong emotions); ‘let it out’ (releasing emotional energy, verbally or physically, in healthy ways); ‘think it out’ (changing cognitions and appraisals about emotional triggers)	In-class completion
Kuosmanen et al (2017)^41^	SPARX-R	To treat the symptoms of mild to moderate depression in adolescents. SPARX-R is framed as a preventative programme; instead of focusing exclusively on depression, SPARX-R is aimed at young people who ‘feel down, stressed or angry’.	CD-ROM accessed on a computer	A designated classroom at school	49 days^b^	7 ‘levels’, each taking 20–30 min to complete	Learning abilities related to emotion regulation (e.g. emotional awareness); mindfulness activities (e.g. breathing, body scan); behavioural activation; CBT activities (e.g. cognitive restructuring)	In-class completion; self-reported number of levels completed
Mrazek et al (2019)^39^	Finding Focus	To help students train their ability to focus and use attention to relate more effectively to thoughts, evaluations and emotions	Internet browser, accessed via computer, tablet or phone	A designated classroom at school	22 days	2.25 h of content; includes four 12 min lessons and daily 4 min exercises	Mindfulness activities (i.e. MBAT); learning and applying attention-based strategies for emotion regulation; anchoring was defined as deciding where you focus; focusing was defined as directing your attention to a specific thing; releasing was defined as letting something go by and not giving it any further attention	In-class completion; monitored electronically through the learning platform
Schnitker et al (2021)^40^	CharacterMe	To engage adolescents in the use of research-informed strategies for building the character strengths of self-control and patience, as well as underlying emotion regulation capacities	Internet browser, mobile app	Wherever the participant chooses	14 days	Participants advised to engage with the app daily for 5–15 min; total 210 min of content	Mood monitoring; mindfulness activities	Monitored electronically through in-app technologies; push notifications

CBT, cognitive–behavioural therapy; CD-ROM, compact disc read-only memory; iTRAC, talking about risk and adolescent choices; MBAT, mindfulness-based attention training; MBSR, mindfulness-based stress reduction.

a. Participants retained access to the intervention following termination of the active intervention period.

b. The duration was longer for some participants due to practical limitations (i.e. missed sessions).

All interventions were self-directed and delivered digitally, although the format varied among studies. Half of the interventions included (*k* = 3) were app based and could be accessed via smartphones whenever and wherever the participant chose.^36,37,40^ The remaining interventions, while self-directed, were delivered during a dedicated intervention session via tablet or computer in a classroom at school.^38,39,41^

### Effects of the interventions

Full details of the interventions and study findings are included in Supplementary File 2. The type of intervention, intervention components, outcomes reported, effect sizes and any significant results are summarised in Table 4.

**Table 4:** Intervention components and outcomes reported

Study: author details	Intervention component					Outcomes reported			Effect (*d*), 95% CI
	Mindfulness, MBSR, MBAT	Mood monitoring	Psychoeducation	Strategy-based learning for ER	Enhancing abilities related to ER	ER	Psychopathology	Related functional outcomes	ER
Hilt and Swords^36^	****	****				**	** [A, IS] **** [D]	**** [W]	0.71, 95% CI [0.17 to 1.00]
Hilt et al^37^	****	****				**	** [D] **** [A]		0.43, 95% CI [0.19 to 0.67]
Houck et al^38^			****	****	****	*		**** [DT]	−0.59, 95% CI [–1.22 to 0.04] [AW]; −0.36, 95% CI –0.81 to 0.10] [AC]; −0.29, 95% CI [–0.82 to 0.24] [CT]; −0.08, 95% CI [–0.67 to 0.50] [CL]
Kuosmanen et al^41^	****		****	****	****	** [ES] **** [CR]	**** [D] **** [A]	**** [WB, C]	0.40, 95% CI [−0.89 to 0.09] [ES]; 0.08, 95% CI [−0.56 to 0.40] [CR]
Mrazek et al^39^	****		****			**		** [PD, SM] **** [S, LS]	0.29, 95% CI [0.16 to 0.42]
Schnitker et al^40^	****	****	****			****		**** [P, SC]	Lack of data

A, anxiety symptoms; AC, access; AW, awareness; C, coping; CL, clarity; CR, cognitive reappraisal; CT, control; D, depressive symptoms; DT, distress tolerance; ER, emotion regulation; ES, expressive suppression; IS, internalising symptoms; LS, life satisfaction; MBAT, mindfulness-based attention training; MBSR, mindfulness-based stress reduction; P, patience; PD, perceived level of demand; S, stress; SC, self-control; SM, stress management; W, worry; WB, well-being.

*, outcome reported but significance not measured; **, significant improvement in outcome found (*P* < 0.05); ***, significant deterioration in outcome found (*P* < 0.05); ****, no significant improvement in outcome found.

#### Interventions targeting antecedent-focused emotion regulation strategies: mindfulness and mood-monitoring

The most common component across most identified interventions was mindfulness – an approach that focuses on observing, describing, acting with awareness, non-judging and non-reactivity to emotional experiences.^42^ A total of four of the five interventions included a mindfulness component (i.e. breathing work, meditation) to support young people in regulating their emotions.^36,37,39–41^ Significant improvements to emotion regulation were observed in three of these interventions,^36,37,39,41^ while the fourth observed no significant improvements in emotion regulation over time.^40^ Despite the significant improvements observed, overall effect sizes were small to moderate (see Table 4). Notably, some adverse effects were observed. In the study by Hilt and colleagues,^37^ a mindfulness-based intervention with a mood-monitoring component was compared with a mood-monitoring-only control. While significant reductions in rumination were observed overall (*t*[69.86] = 3.53, *P* < 0.001), a quarter of adolescents in the mindfulness condition experienced clinically significant worsening of rumination at the end of the intervention period.^37^

Psychopathology was included as an outcome of just two of the interventions across three studies. The first intervention was tested across a cohort study^36^ and a pilot RCT;^37^ it included mindfulness and mood-monitoring components. Overall, although the intervention reduced anxiety symptoms in both studies, this reduction was non-significant in the pilot RCT.^37^ In contrast, while the authors observed a significant reduction in depressive symptoms in the pilot RCT,^37^ there was no significant impact of the intervention on depressive symptoms in the cohort study.^36^

#### Interventions targeting antecedent-focused RCTs (strategies: problem-solving and behavioural avoidance)

In addition to mindfulness activities, the intervention by Mrazek and colleagues^39^ included exercises to target other antecedent-focused emotion regulation strategies, such as problem-solving or behavioural avoidance. In this cohort study, the intervention was found to significantly improve emotion regulation in adolescents but the effect size was small overall (*d* = 0.29). After participating in the intervention, adolescents also showed significant changes in perceived stress management. Despite this, there were non-significant changes in actual stress and overall life satisfaction among adolescents.^39^ Psychopathology was not included as an outcome in this study.

#### Interventions targeting multiple RCTs (strategies and related skills (e.g. emotional awareness))

Two interventions identified^38,41^ had a wider focus, targeting several different antecedent- and response-focused emotion regulation strategies (e.g. acceptance, expressive suppression). The first^38^ included activities to develop the skills related to emotion regulation, such as emotional awareness and emotion controllability beliefs. This intervention was tested in a pilot RCT with a wait-list control, and was found to be beneficial in improving emotion regulation overall. Participants who completed this intervention perceived themselves as having better emotional competence and endorsed greater use of the emotion regulation strategies targeted by the intervention. Participants reported greater belief that emotions can be changed, greater awareness of their emotions, self-efficacy for managing emotions and perceived access to emotion regulation strategies. There were unexpected effects for behavioural measures of distress tolerance: participants who completed the intervention persisted for less time on tasks and showed reduced distress tolerance compared with wait-list controls. Psychopathology was not included as an outcome in this study.

The second intervention^41^ targeted a broad spectrum of emotion regulation strategies and abilities through a cognitive behavioural therapy (CBT) and behavioural activation approach. Although one module was dedicated to emotion regulation, different strategies and abilities relevant to emotion regulation were included throughout (i.e. problem-solving, cognitive reappraisal, emotional awareness). The intervention was tested in a cluster RCT and demonstrated mixed effects for improving emotion regulation among participants. It was shown to be significant in improving expressive suppression when compared with a wait-list control. However, a non-significant decrease in cognitive reappraisal was observed. There were no significant intervention effects observed for either depressive or anxiety symptoms.

### Feasibility and acceptability of the interventions

The acceptability and feasibility of these interventions are illustrated in Table 5. Because most interventions were in the earlier stages of their development, for many studies, understanding the acceptability and feasibility of the intervention was very much the focus.

**Table 5:** Acceptability of the interventions

Study: author details	Measurement	Findings	Adverse events?	Conclusions
Hilt and Swords^36^	Feedback ratings about: Ease of use Clarity of instruction Would they recommend the app to other young people? Parent and adolescent report Adverse event monitoring	Mean ratings (on a 7-point scale): Ease of use, 6.11 Clarity of instructions, 5.40 77.4% said ‘Yes’, that other young people would find the app useful. 73.2% of parents reported that their children benefited from using the app.	None	The majority of adolescents found the app easy to use, and instructions for the mindfulness exercises clear and understandable. Most adolescents thought other young people would find this app helpful. Parents agreed that the app had benefits for adolescents.
Hilt et al^37^	Adverse event monitoring References to previous pilot study^36^	Few adverse events reported to demonstrate the acceptability of the app.	9 reported, 4 related to the study; all four of these events involved increased awareness of negative feelings during the intervention period. Two participants reported this in each condition; thus, it may be related to monitoring negative emotions	App is acceptable for adolescents. Adverse events involved an increase in awareness of negative emotions.
Houck et al^38^	Semi-structured interviews (*n* = 11) to assess comprehension and engagement with the content Feedback ratings	Mean ratings (on a 5-point scale): Ease of use, 4.7 Information helpfulness, 4.3 Overall experience, 4.3 Qualitative interview data revealed high acceptability.	Not reported	The intervention was highly acceptable to adolescents. The majority of adolescents found the intervention easy to use, that it provided useful information and had a positive overall experience.
Kuosmanen et al^41^	Structured implementation questionnaire Findings in relation to four elements of acceptability are reported in this study: Number of levels completed (self-reported) Overall satisfaction score (on a scale of 1 to 10) Perceived helpfulness Extent of practising the skills taught in the programme	Participants completed on average 5.3 levels (76%) of SPARX-R. SPARX-R received an overall satisfaction score of 6.0 (s.d. 2.76) out of 10. The programme was rated most helpful in terms of confronting problems and recognising negative thoughts. Approximately 40% reported SPARX-R being helpful in relation to managing and expressing emotions. The programme was rated least helpful in terms of changing behaviour. Of the skills and strategies taught in SPARX-R, the most frequently practised techniques were cognitive behavioural techniques; 65.2% reported having practised recognising personal negative thinking patterns. The least utilised techniques were activity scheduling and relaxation techniques.	Not reported	The intervention had a good overall satisfaction score and level of completion by participants. The intervention was most helpful in confronting problems and recognising negative thoughts. The intervention was least helpful in changing behaviour. The most frequently practised intervention techniques were cognitive behavioural techniques; the least utilised techniques were activity scheduling and relaxation techniques.

Most studies (*k* = 4) measured intervention acceptability among adolescents; to do so, various quantitative and qualitative methods were employed. Overall, the authors commented that their interventions were acceptable for use among adolescents, in keeping with previous evidence.^22,43^ Many adolescents felt that the interventions were simple to use and that the exercises were clear, helpful and easy to understand. One study also measured parents’ perceptions, and found that they similarly reported the beneficial impact of the intervention on their children.^36^

In one of the studies, differences were observed in terms of the extent to which different intervention components were perceived as helpful.^41^ Participants reported that the intervention was most helpful in confronting problems and recognising negative thoughts, but least helpful in changing behaviour. In this particular intervention, the least utilised activities were relaxation and mindfulness techniques.

Importantly, some participants had negative experiences with some of the interventions, either finding them not helpful at all or reporting that they had negatively impacted their mental health or ability to regulate their emotions. One study reported four adverse events among their participants;^37^ all four events involved increased awareness of negative thoughts and feelings during the intervention period. Two participants reported this in each condition (mindfulness + mood-monitoring versus mood-monitoring alone); this may be related to monitoring negative emotions.

## Discussion

**Fig. 3:**
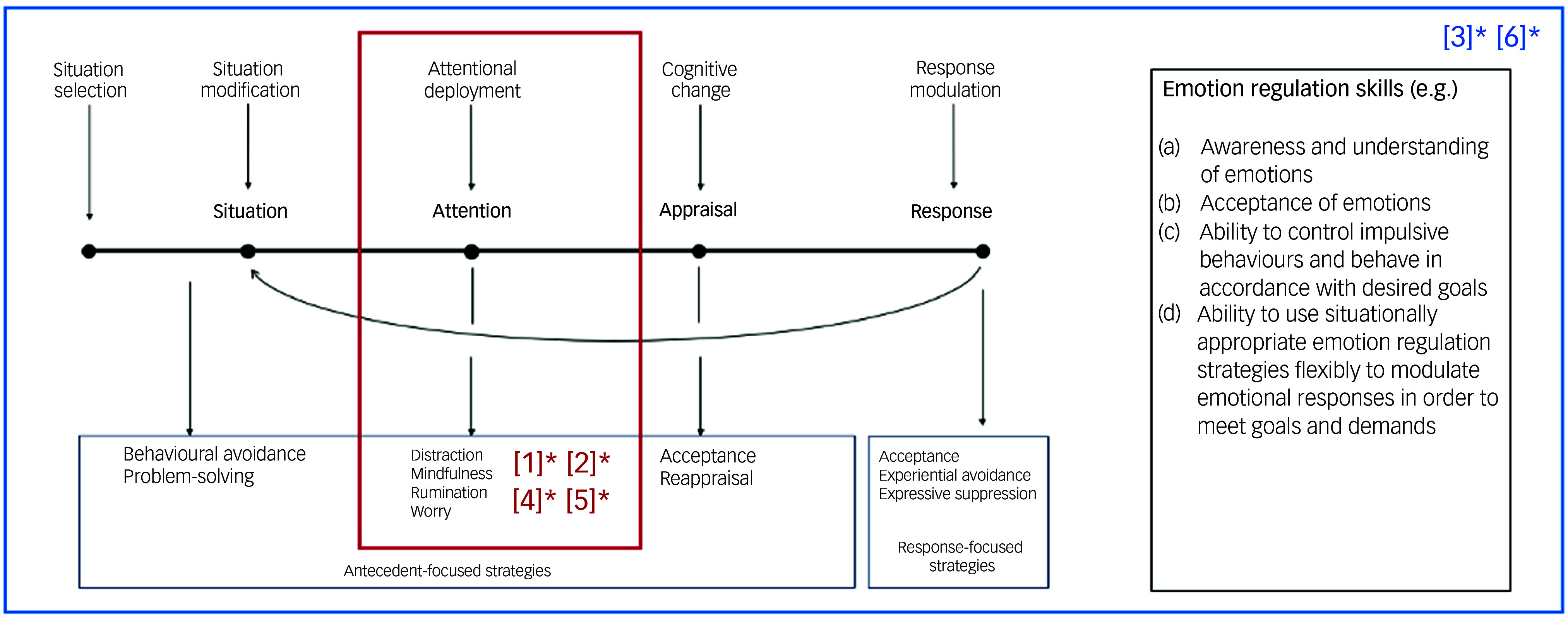
Model of emotion regulation intervention targets. This figure shows Gross’s (1998) Process Model of Emotion Regulation. Examples of related emotion regulation skills are listed alongside. The included interventions are numbered from one to six. [1] Hilt & Swords, 2021, [2] Hilt et al, 2023, [3] Houck et al, 2022, [4] Mrazek et al, 2019, [5] Schnitker et al, 2021, [6] Kuosmanen et al, 2017. Those shown in the red box [1–2, 4–5] included intervention components that primarily targeted attention processes in emotion regulation. Those shown in blue [3, 6] targeted each emotion regulation process, from situation selection to response modulation, as well as related emotion regulation skills shown in the figure. NB: []*significant improvement in emotion regulation found in the present systematic review (*P* < 0.05).

The aim of this systematic review was to synthesise evidence on current self-directed, digital interventions that target emotion regulation, and to explore their benefits and acceptability among adolescents (aged 11–18 years). Overall, the evidence base was small. Just six papers were identified, which measured the effects of a total of five brief interventions for emotion regulation. There was wide clinical and methodological diversity across interventions and studies; each of the interventions included different components to target emotion regulation. These components have been mapped onto the Process Model of Emotion Regulation^12^ to better illustrate the diversity in approaches to targeting emotion regulation in adolescence (Fig. 3). Although Gross’s Process Model of Emotion Regulation^12^ suggests dynamic variability in the consequences of each of the proposed emotion regulation strategies (e.g. cognitive reappraisal), in practice, researchers and clinicians alike have tended to interpret these as either adaptive (e.g. mindfulness, acceptance) or maladaptive (e.g. withdrawal, suppression^44^). This has similarly directed intervention efforts, with the focus often being on developing interventions aimed at increasing the use of ‘adaptive’ strategies and/or decreasing the use of ‘maladaptive’ strategies.

This was reflected in the interventions identified in this review. The majority focused on increasing the use of adaptive strategy mindfulness to improve emotion regulation. Mindfulness-based interventions have gathered increasing attention within the field,^45^ and are increasingly employed in schools to support adolescents with their emotional well-being and mental health.^46^ Although mindfulness was offered as a component across four of the five identified digital interventions, findings on its benefits were mixed. Despite some significant improvements in emotion regulation being observed, effect sizes were small overall. One study also reported that participants engaged with mindfulness techniques the least when compared with other intervention activities.^41^ Of particular note in one study^37^ was the finding that a quarter of adolescents who completed the mindfulness-based intervention experienced clinically significant worsening of rumination. This would align with existing evidence that mindfulness is not always a helpful strategy, particularly when applied universally to the adolescent population.^47^

This study also reported four adverse events among their participants; all four events involved increased awareness of negative feelings during the intervention period, supporting previous findings in adults that mood monitoring, in the absence of adequate strategies to process the emotions identified, can sometimes be harmful rather than helpful.^48,49^ Indeed, there is a growing consensus that the adaptiveness of any given emotion regulation strategy is context dependent rather than universal and depends on the appropriateness of the strategy to the specific situation in which it is used.^44^ There has been increased evidence that the use and functional benefits of any specific type of emotion regulation strategy will tend to vary across people and situations and, by extension, the most effective use of emotion regulation strategies is likely to be one that is most flexible.^44,50^ Accordingly, interventions such as those proposed by Houck and colleagues^38^ and Kuosmanen and colleagues,^41^ which include activities to develop the related skills required to access certain strategies and use them flexibly, may be the most appropriate approach to supporting adolescents with emotion regulation and their mental health.

Few studies have measured the impact of targeting emotion regulation on mental health outcomes. The impact of these interventions on adolescent psychopathology was measured in just three studies, two of which investigated the same intervention.^36,37^ Overall findings were mixed: while reductions in symptoms of anxiety and depression were observed, these were non-significant across studies. Given the growing evidence on the importance of emotion regulation for psychopathology in adolescence,^19^ its omission as an outcome in most studies is surprising and limits current attempts to better understand its role in supporting adolescents. However, this may reflect the fact that most interventions were in the earlier stages of their development and, therefore, understanding the feasibility of the interventions in targeting emotion regulation was the primary focus for most studies.^51^ Greater research is needed to understand the effects of targeting emotion regulation through self-directed digital interventions on adolescent psychopathology.

### Clinical implications

This review identified a total of 6 studies that investigated the effects of 5 self-directed, digital interventions available to adolescents (aged 11–18 years). Despite large methodological and clinical heterogeneity, preliminary evidence from these studies suggests that these interventions showed benefits for emotion regulation. The self-directed and digital format was also highly feasible and acceptable to adolescents, a finding that is in keeping with previous research on digital interventions for young people.^43^ Although the evidence base was small, this review highlights some important considerations for researchers and practitioners working to support adolescents with their mental health.

Primarily, although most of the interventions employed mindfulness as a strategy to improve emotion regulation, this was not a universally acceptable or beneficial approach. Given the deluge of literature on mindfulness-based interventions, such a finding suggests that there is a need to look elsewhere for additional solutions to support young people with their emotion regulation and mental health. One such recommendation would be to focus on developing research into other emotion regulation strategies that receive arguably less attention (e.g. cognitive reappraisal) and the related skills required to access these strategies and use them flexibly. Individual, social, cultural and environmental factors are known to be highly influential in the success of different emotion regulation strategies.^44^ As such, the focus of interventions should move beyond teaching adolescents different strategies, to also equipping them with the skills they need to apply these confidently (e.g. emotional awareness, self-efficacy). A further key finding of note from this review was the strong feasibility and acceptability of the self-directed digital format for delivering this type of intervention to adolescents. Digital health is a growing field, and provides opportunities to broaden access to care in a cost-effective and sustainable manner. Particularly among adolescents, this is known to be a highly acceptable format for the delivery of mental health interventions.^43^

### Limitations of this review

The findings of this review should be interpreted with caution due to several limitations. Primarily, the lack of studies identified limits any conclusions that could be drawn. Despite the planned focus of this review on psychopathology, as it transpired, just three studies measured this as an outcome and findings were mixed. Moreover, only symptoms of anxiety and depression were measured, making it difficult to draw conclusions about the effects of emotion regulation as a transdiagnostic mechanism. There was also significant clinical and methodological heterogeneity among identified studies, meaning that direct comparison of interventions was not possible, nor making conclusions about those most effective in improving emotion regulation and psychopathology. However, we argue that insight can be gleaned from understanding the different approaches currently taken to improve emotion regulation in adolescents and their effects within individual studies, but acknowledge that, with the lack of available evidence, no definitive conclusions should be drawn about the effectiveness of any individual approach in comparison with another.

Only 50% of studies were dually screened at title and abstract stage. Although rater pairs showed strong agreement in those articles that were screened, the lack of 100% dual screening may have contributed to the decreased number of identified studies.

There was considerable variation across the measures used to capture change in emotion regulation, as expected given the large variation in the different conceptualisations of emotion regulation and its subsequent measurement.^52^ Although some validated measures exist, these are not employed consistently across studies of emotion regulation, meaning that it is difficult to compare the effects of different interventions. Similarly, functional outcomes such as academic achievement or life satisfaction were different across all of the included studies and thus could not be compared.

There were further limitations within the studies included in this review. The quality of the studies was limited, and many of the interventions were at the early stages of testing. Most studies were pre- to post-cohort studies and did not include a comparison group, which contributed to the increased risk of bias. Similarly, many of the included studies had short follow-up time periods, meaning that any change in effects observed over time was not captured here. Furthermore, although the participants included in these studies were ethnically diverse, most were conducted in the USA, limiting conclusions that can be drawn about the benefits of these interventions globally. As such, many findings that were reported in this review should be interpreted in light of these shortcomings.

### Suggestions for future research

As the prevalence of mental health problems among adolescents increases, more research must be done to determine the impact of self-directed digital interventions targeting transdiagnostic mechanisms like emotion regulation, particularly when applied at scale to support adolescents with their mental health. Recommendations from this research echo a common suggestion within the emotion regulation literature – to better refine and clarify the concept of emotion regulation as it applies to research and practice. In this review, several different measures of emotion regulation were employed, dependent on the authors’ understanding of emotion regulation as a concept to be targeted in an intervention. The lack of conceptual clarity surrounding emotion regulation, and the way in which its change can be interpreted or measured, mean that it is difficult to compare and synergise emotion regulation interventions and illuminate those components that are most beneficial in improving emotion regulation and mental health. Clarifying the definition of emotion regulation is an essential step to a more synchronous approach in the field, and ultimately to better understanding of how this mechanism can be targeted to support adolescents with their mental health.

## Supporting information

Thomson et al. supplementary material 1 Thomson et al. supplementary material

Thomson et al. supplementary material 2 Thomson et al. supplementary material

Thomson et al. supplementary material 3 Thomson et al. supplementary material

## Data Availability

Data availability is not applicable to this article because no new data were created or analysed in this study.

## Supplementary material

The supplementary material is available online at https://doi.org/10.1192/bjo.2025.10888
