# Supplementary material for: Self-directed digital interventions for the improvement of emotion regulation – acceptability and feasibility for adolescents: systematic review
Source: BJPsych Open. 2025 Nov 6;11(6):e270. doi: 10.1192/bjo.2025.10888 (PMC12641403; doi:10.1192/bjo.2025.10888)
Supplement: Thomson et al. supplementary material 2 — Thomson et al. supplementary material [file S2056472425108880sup002.pdf]

PRISMA 2020 for Abstracts Checklist

Section and Topic	Item #	Checklist item	Reported (Yes/No)
**TITLE**			
Title	1	Identify the report as a systematic review.	Yes
**BACKGROUND**			
Objectives	2	Provide an explicit statement of the main objective(s) or question(s) the review addresses.	Yes
**METHODS**			
Eligibility criteria	3	Specify the inclusion and exclusion criteria for the review.	Yes
Information sources	4	Specify the information sources (e.g. databases, registers) used to identify studies and the date when each was last searched.	Yes
Risk of bias	5	Specify the methods used to assess risk of bias in the included studies.	Yes
Synthesis of results	6	Specify the methods used to present and synthesise results.	Yes
**RESULTS**			
Included studies	7	Give the total number of included studies and participants and summarise relevant characteristics of studies.	Yes
Synthesis of results	8	Present results for main outcomes, preferably indicating the number of included studies and participants for each. If meta-analysis was done, report the summary estimate and confidence/credible interval. If comparing groups, indicate the direction of the effect (i.e. which group is favoured).	Yes
**DISCUSSION**			
Limitations of evidence	9	Provide a brief summary of the limitations of the evidence included in the review (e.g. study risk of bias, inconsistency and imprecision).	Yes
Interpretation	10	Provide a general interpretation of the results and important implications.	Yes
**OTHER**			
Funding	11	Specify the primary source of funding for the review.	No
Registration	12	Provide the register name and registration number.	Yes

From: Page MJ, McKenzie JE, Bossuyt PM, Boutron I, Hoffmann TC, Mulrow CD, et al. The PRISMA 2020 statement: an updated guideline for reporting systematic reviews. BMJ 2021;372:n71. doi: 10.1136/bmj.n71. This work is licensed under CC BY 4.0. To view a copy of this license, visit https://creativecommons.org/licenses/by/4.0/

PRISMA 2020 Checklist APPENDIX S2: PRISMA CHECKLIST

Section and Topic	Item #	Checklist item	Location where item is reported
**TITLE**			
Title	1	Identify the report as a systematic review.	Title Page
**ABSTRACT**			
Abstract	2	See the PRISMA 2020 for Abstracts checklist.	Page 2<br>Supp Mat
**INTRODUCTION**			
Rationale	3	Describe the rationale for the review in the context of existing knowledge.	Page 3-5
Objectives	4	Provide an explicit statement of the objective(s) or question(s) the review addresses.	Page 5
**METHODS**			
Eligibility criteria	5	Specify the inclusion and exclusion criteria for the review and how studies were grouped for the syntheses.	Table 1<br>Page 7-8
Information sources	6	Specify all databases, registers, websites, organisations, reference lists and other sources searched or consulted to identify studies. Specify the date when each source was last searched or consulted.	Page 6-7
Search strategy	7	Present the full search strategies for all databases, registers and websites, including any filters and limits used.	Supp Mat
Selection process	8	Specify the methods used to decide whether a study met the inclusion criteria of the review, including how many reviewers screened each record and each report retrieved, whether they worked independently, and if applicable, details of automation tools used in the process.	Page 7
Data collection process	9	Specify the methods used to collect data from reports, including how many reviewers collected data from each report, whether they worked independently, any processes for obtaining or confirming data from study investigators, and if applicable, details of automation tools used in the process.	Page 7-8
Data items	10a	List and define all outcomes for which data were sought. Specify whether all results that were compatible with each outcome domain in each study were sought (e.g. for all measures, time points, analyses), and if not, the methods used to decide which results to collect.	Table 1<br>Page 8-9
	10b	List and define all other variables for which data were sought (e.g. participant and intervention characteristics, funding sources). Describe any assumptions made about any missing or unclear information.	Page 8
Study risk of bias assessment	11	Specify the methods used to assess risk of bias in the included studies, including details of the tool(s) used, how many reviewers assessed each study and whether they worked independently, and if applicable, details of automation tools used in the process.	Page 8-9
Effect measures	12	Specify for each outcome the effect measure(s) (e.g. risk ratio, mean difference) used in the synthesis or presentation of results.	N/A
Synthesis methods	13a	Describe the processes used to decide which studies were eligible for each synthesis (e.g. tabulating the study intervention characteristics and comparing against the planned groups for each synthesis (item #5)).	Page 9
	13b	Describe any methods required to prepare the data for presentation or synthesis, such as handling of missing summary statistics, or data conversions.	N/A
	13c	Describe any methods used to tabulate or visually display results of individual studies and syntheses.	Page 8
	13d	Describe any methods used to synthesize results and provide a rationale for the choice(s). If meta-analysis was performed, describe the model(s), method(s) to identify the presence and extent of statistical heterogeneity, and software package(s) used.	Page 8-9
	13e	Describe any methods used to explore possible causes of heterogeneity among study results (e.g. subgroup analysis, meta-regression).	N/A
	13f	Describe any sensitivity analyses conducted to assess robustness of the synthesized results.	N/A
Reporting bias	14	Describe any methods used to assess risk of bias due to missing results in a synthesis (arising from reporting biases).	N/A

APPENDIX S2: PRISMA CHECKLIST

Section and Topic	Item #	Checklist item	Location where item is reported
assessment			
Certainty assessment	15	Describe any methods used to assess certainty (or confidence) in the body of evidence for an outcome.	N/A
**RESULTS**			
Study selection	16a	Describe the results of the search and selection process, from the number of records identified in the search to the number of studies included in the review, ideally using a flow diagram.	Figure 2<br>Page 9
	16b	Cite studies that might appear to meet the inclusion criteria, but which were excluded, and explain why they were excluded.	N/A
Study characteristics	17	Cite each included study and present its characteristics.	Table 2<br>Page 9-10
Risk of bias in studies	18	Present assessments of risk of bias for each included study.	Table 2<br>Page 10<br>Supp Mat
Results of individual studies	19	For all outcomes, present, for each study: (a) summary statistics for each group (where appropriate) and (b) an effect estimate and its precision (e.g. confidence/credible interval), ideally using structured tables or plots.	Table 4
Results of syntheses	20a	For each synthesis, briefly summarise the characteristics and risk of bias among contributing studies.	Page 9-10
	20b	Present results of all statistical syntheses conducted. If meta-analysis was done, present for each the summary estimate and its precision (e.g. confidence/credible interval) and measures of statistical heterogeneity. If comparing groups, describe the direction of the effect.	Table 4<br>Page 11-14
	20c	Present results of all investigations of possible causes of heterogeneity among study results.	N/A
	20d	Present results of all sensitivity analyses conducted to assess the robustness of the synthesized results.	N/A
Reporting biases	21	Present assessments of risk of bias due to missing results (arising from reporting biases) for each synthesis assessed.	N/A
Certainty of evidence	22	Present assessments of certainty (or confidence) in the body of evidence for each outcome assessed.	N/A
**DISCUSSION**			
Discussion	23a	Provide a general interpretation of the results in the context of other evidence.	Page 14-16
	23b	Discuss any limitations of the evidence included in the review.	Page 16-17
	23c	Discuss any limitations of the review processes used.	Page 16-17
	23d	Discuss implications of the results for practice, policy, and future research.	Page 16-18
**OTHER INFORMATION**			
Registration and protocol	24a	Provide registration information for the review, including register name and registration number, or state that the review was not registered.	Page 2, 6
	24b	Indicate where the review protocol can be accessed, or state that a protocol was not prepared.	Page 6
	24c	Describe and explain any amendments to information provided at registration or in the protocol.	Page 6
Support	25	Describe sources of financial or non-financial support for the review, and the role of the funders or sponsors in the review.	Title page
Competing interests	26	Declare any competing interests of review authors.	Title page

APPENDIX S2: PRISMA CHECKLIST

Section and Topic	Item #	Checklist item	Location where item is reported
Availability of data, code and other materials	27	Report which of the following are publicly available and where they can be found: template data collection forms; data extracted from included studies; data used for all analyses; analytic code; any other materials used in the review.	Title page

From: Page MJ, McKenzie JE, Bossuyt PM, Boutron I, Hoffmann TC, Mulrow CD, et al. The PRISMA 2020 statement: an updated guideline for reporting systematic reviews. BMJ 2021;372:n71. doi: 10.1136/bmj.n71

For more information, visit: http://www.prisma-statement.org/

SUPPLEMENTARY FILE 1

Appendix S1: Search Strategy

Database: Ovid PsycINFO 1806 to present
Search Strategy:

----

1 (adolescen* or juvenil* or youth* or teen* or pubescen* or pediatric* or paediatric* or "high school" or "secondary school" or school* or "young people" or "young person*" or "young adult*" or "young man" or "young men" or "young woman" or "young women" or student*).ti,ab

2 (emotion* AND (regulat* or dysregulat* or generat* OR modulat* OR adapt* OR cop* OR react* OR arous* OR avoid* OR behavio* OR suppress* OR ruminat*)).,ti,ab

3 (affect AND (regulat* or dysregulat* or generat* OR modulat* OR adapt* OR cop* OR react* OR arous* OR avoid* OR behavio* OR suppress* OR ruminat*)).ti,ab

4 ("self-concept" OR "mood regulat*" OR "situation selection" OR "situation modification" OR "attentional deployment" OR "response modulation" OR "problem focused coping" OR "impulsivity" OR "hyper-reactivity" OR "anger regulation" OR "experiential avoidance" OR "expressive suppression" OR "thought suppression" OR "ruminat*" OR "emotion regulation questionnaire" OR "Difficulties in Emotion Regulation Scale" OR "emotion regulation strategy").ti,ab

5 2 or 3 or 4

6 (treat* or interven* or "early intervention" or "self-directed intervention" or "self-directed" or "public health").ti,ab.

7 phon* or mobile* or "smart-phone*" or device* or computer* or tablet* or "device-based" or "app-based" or apps or app or "sensor*" or "social media").ti,ab.

8 ((digital or virtual or online or interactive) and (health* or intervention or support or platform*)).ti,ab.

9 ("eHealth" or "e-health" or "m-health" or mHealth or "tele-health" or telehealth).ti,ab.

10 7 or 8 or 9

11 1 and 5 and 6 and 10

12 limit 11 to (peer reviewed journal and human and english language and (childhood <birth to 12 years> or adolescence <13 to 17 years>) and ("0100 journal" or "0110 peer-reviewed journal") and journal article and english and human and yr="1995 -Current")

Database: PubMed MEDLINE

Search Strategy:

----

1 adolescen*[Title/Abstract] OR juvenil*[Title/Abstract] OR youth*[Title/Abstract] OR teen*[Title/Abstract] OR pubescen*[Title/Abstract] OR pediatric*[Title/Abstract] OR paediatric*[Title/Abstract] OR "high school"[Title/Abstract] OR "secondary school"[Title/Abstract] OR school*[Title/Abstract] OR "young people"[Title/Abstract] OR "young person*"[Title/Abstract] OR "young adult*"[Title/Abstract] OR "young man"[Title/Abstract] OR "young men"[Title/Abstract] OR "young woman"[Title/Abstract] OR "young women"[Title/Abstract] OR student*[Title/Abstract]

2 emotion*[Title/Abstract] AND (regulat*[Title/Abstract] OR dysregulat*[Title/Abstract] OR generat*[Title/Abstract] OR modulat*[Title/Abstract] OR adapt*[Title/Abstract] OR react*[Title/Abstract] OR arous*[Title/Abstract] OR avoid*[Title/Abstract] OR behavio*[Title/Abstract] OR suppress*[Title/Abstract] OR ruminat*[Title/Abstract])

3 affect[Title/Abstract] AND (regulat*[Title/Abstract] OR dysregulat*[Title/Abstract] OR generat*[Title/Abstract] OR modulat*[Title/Abstract] OR adapt*[Title/Abstract] OR react*[Title/Abstract] OR arous*[Title/Abstract] OR avoid*[Title/Abstract] OR behavio*[Title/Abstract] OR suppress*[Title/Abstract] OR ruminat*[Title/Abstract])

4 "self-concept"[Title/Abstract] OR "mood regulat*"[Title/Abstract] OR "situation selection"[Title/Abstract] OR "situation modification"[Title/Abstract] OR "attentional deployment"[Title/Abstract] OR "response modulation"[Title/Abstract] OR "problem focused coping"[Title/Abstract] OR "impulsivity"[Title/Abstract] OR "hyper-reactivity"[Title/Abstract] OR "anger regulation"[Title/Abstract] OR "experiential avoidance"[Title/Abstract] OR "expressive suppression"[Title/Abstract] OR "thought suppression"[Title/Abstract] OR "ruminat*"[Title/Abstract] OR "emotion regulation questionnaire"[Title/Abstract] OR "Difficulties in Emotion Regulation Scale"[Title/Abstract] OR "emotion regulation strategy"[Title/Abstract]

5 #2 OR #3 OR #4

6 treat*[Title/Abstract] OR interven*[Title/Abstract] OR "early intervention"[Title/Abstract] OR "self-directed intervention"[Title/Abstract] OR "self-directed"[Title/Abstract] OR "public health"[Title/Abstract]

7 phon*[Title/Abstract] OR mobile*[Title/Abstract] OR "smart-phone*"[Title/Abstract] OR device*[Title/Abstract] OR computer*[Title/Abstract] OR tablet*[Title/Abstract] OR "device-based"[Title/Abstract] OR "app-based"[Title/Abstract] OR apps[Title/Abstract] OR app[Title/Abstract] OR "sensor*"[Title/Abstract] OR "social media"[Title/Abstract]

8	((digital[Title/Abstract] OR virtual[Title/Abstract] OR online[Title/Abstract] OR interactive[Title/Abstract]) AND (health*[Title/Abstract] OR intervention[Title/Abstract] OR support[Title/Abstract] OR platform*[Title/Abstract]))
9	"eHealth"[Title/Abstract] OR "e-health"[Title/Abstract] OR "m-health"[Title/Abstract] OR mHealth[Title/Abstract] OR "tele-health"[Title/Abstract] OR telehealth[Title/Abstract]
10	#7 OR #8 OR #9
11	#1 and #5 and #6 and #10
**12**	**#1 and #5 and #6 and #10 Filters: Humans, English, MEDLINE, from 1995 – 2023**

Database: Scopus

Search Strategy:

(TITLE-ABS-KEY (adolescen* OR juvenil* OR youth* OR teen* OR pubescen* OR pediatric* OR paediatric* OR "high school" OR "secondary school" OR school* OR "young people" OR "young person*" OR "young adult*" OR "young man" OR "young men" OR "young woman" OR "young women" OR student*) AND TITLE-ABS-KEY ((emotion* AND (regulat* OR dysregulat* OR generat* OR modulat* OR adapt* OR cop* OR react* OR arous* OR avoid* OR behavio* OR suppress* OR ruminat*)) OR (affect AND (regulat* OR dysregulat* OR generat* OR modulat* OR adapt* OR cop* OR react* OR arous* OR avoid* OR behavio* OR suppress* OR ruminat*)) OR (self-concept OR "mood regulat*" OR "situation selection" OR "situation modification" OR "attentional deployment" OR "response modulation" OR "problem focused coping" OR impulsivity OR hyper-reactivity OR "anger regulation" OR "experiential avoidance" OR "expressive suppression" OR "thought suppression" OR ruminat* OR "emotion regulation questionnaire" OR "Difficulties in Emotion Regulation Scale" OR "emotion regulation strategy")) AND TITLE-ABS-KEY (treat* OR interven* OR "early intervention" OR "self-directed intervention" OR "self-directed" OR "public health") AND TITLE-ABS-KEY ((phon* OR mobile* OR "smart-phone*" OR device* OR computer* OR tablet* OR "device-based" OR "app-based" OR apps OR app OR "sensor*" OR "social media") OR ((digital OR virtual OR online OR interactive) AND (health* OR intervention OR support OR platform*)) OR ("eHealth" OR "e-health" OR "m-health" OR mhealth OR "tele-health" OR telehealth))) AND ORIG-LOAD-DATE AFT 20230203 AND (LIMIT-TO (SUBJAREA , "MEDI") OR LIMIT-TO (SUBJAREA , "SOCI") OR LIMIT-TO (SUBJAREA , "MULT") OR LIMIT-TO (SUBJAREA , "PSYC") OR LIMIT-TO (SUBJAREA , "NURS") OR LIMIT-TO (SUBJAREA , "HEAL")) AND (LIMIT-TO (DOCTYPE , "ar")) AND (LIMIT-TO (EXACTKEYWORD , "Human") OR LIMIT-TO (EXACTKEYWORD , "Humans") OR LIMIT-TO (EXACTKEYWORD , "Adolescent")) AND (LIMIT-TO (LANGUAGE , "English")) AND (EXCLUDE (SUBJAREA , "MEDI") OR EXCLUDE (SUBJAREA , "NEUR") OR EXCLUDE (SUBJAREA , "BIOC") OR EXCLUDE (SUBJAREA , "ARTS") OR EXCLUDE (SUBJAREA , "ENVI") OR EXCLUDE (SUBJAREA , "PHAR")) AND (EXCLUDE (SUBJAREA , "AGRI") OR EXCLUDE (SUBJAREA , "BUSI") OR EXCLUDE (SUBJAREA , "COMP") OR EXCLUDE (SUBJAREA , "DENT") OR EXCLUDE (SUBJAREA , "ENGI")) AND (EXCLUDE (SUBJAREA , "VETE"))

Database: Global Health 1973

Search Strategy:

----

1 (adolescen* or juvenil* or youth* or teen* or pubescen* or pediatric* or paediatric* or "high school" or "secondary school" or school* or "young people" or "young person*" or "young adult*" or "young man" or "young men" or "young woman" or "young women" or student*).ti,ab

2 (emotion* AND (regulat* or dysregulat* or generat* OR modulat* OR adapt* OR cop* OR react* OR arous* OR avoid* OR behavio* OR suppress* OR ruminat*)).,ti,ab

3 (affect AND (regulat* or dysregulat* or generat* OR modulat* OR adapt* OR cop* OR react* OR arous* OR avoid* OR behavio* OR suppress* OR ruminat*)).,ti,ab

4 ("self-concept" OR "mood regulat*" OR "situation selection" OR "situation modification" OR "attentional deployment" OR "response modulation" OR "problem focused coping" OR "impulsivity" OR "hyper-reactivity" OR "anger regulation" OR "experiential avoidance" OR "expressive suppression" OR "thought suppression" OR "ruminat*" OR "emotion regulation questionnaire" OR "Difficulties in Emotion Regulation Scale" OR "emotion regulation strategy").ti,ab

5 2 or 3 or 4

6 (treat* or interven* or "early intervention" or "self-directed intervention" or "self-directed" or "public health").ti,ab.

7 phon* or mobile* or "smart-phone*" or device* or computer* or tablet* or "device-based" or "app-based" or apps or app or "sensor*" or "social media").ti,ab.

8 ((digital or virtual or online or interactive) and (health* or intervention or support or platform*)).ti,ab.

9 ("eHealth" or "e-health" or "m-health" or mHealth or "tele-health" or telehealth).ti,ab.

10 7 or 8 or 9

11 1 and 5 and 6 and 10

12 limit 11 to (english language and english and (journal or journal article) and yr="1995 -Current")

Database: EBSCO CINAHL

Search Strategy:

----

1 AB adolescen* or juvenil* or youth* or teen* or pubescen* or pediatric* or paediatric* or "high school" or "secondary school" or school* or "young people" or "young person*" or "young adult*" or "young man" or "young men" or "young woman" or "young women" or student*

2 AB emotion* AND (regulat* or dysregulat* or generat* OR modulat* OR adapt* OR cop* OR react* OR arous* OR avoid* OR behavio* OR suppress* OR ruminat*)

3 AB affect AND (regulat* or dysregulat* or generat* OR modulat* OR adapt* OR cop* OR react* OR arous* OR avoid* OR behavio* OR suppress* OR ruminat*)

4 AB "self-concept" OR "mood regulat*" OR "situation selection" OR "situation modification" OR "attentional deployment" OR "response modulation" OR "problem focused coping" OR "impulsivity" OR "hyper-reactivity" OR "anger regulation" OR "experiential avoidance" OR "expressive suppression" OR "thought suppression" OR "ruminat*" OR "emotion regulation questionnaire" OR "Difficulties in Emotion Regulation Scale" OR "emotion regulation strategy"

5 S2 or S3 or S4

6 AB treat* or interven* or "early intervention" or "self-directed intervention" or "self-directed" or "public health"

7 AB phon* or mobile* or "smart-phone*" or device* or computer* or tablet* or "device-based" or "app-based" or apps or app or "sensor*" or "social media"

8 AB (digital or virtual or online or interactive) and (health* or intervention or support or platform*))

9 AB "eHealth" or "e-health" or "m-health" or mHealth or "tele-health" or telehealth

10 S7 or S8 or S9

11 S1 and S5 and S6 and S10

Limiters - Published Date: 19950101-; English Language; Peer Reviewed; Research Article; Human; Publication Type: Journal Article; Age Groups: Child: 6-12 years, Adolescent: 13-18 years; Language: English

Database: EBSCO ERIC

Search Strategy:

----

1 AB adolescen* or juvenil* or youth* or teen* or pubescen* or pediatric* or paediatric* or "high school" or "secondary school" or school* or "young people" or "young person*" or "young adult*" or "young man" or "young men" or "young woman" or "young women" or student*

2 AB emotion* AND (regulat* or dysregulat* or generat* OR modulat* OR adapt* OR cop* OR react* OR arous* OR avoid* OR behavio* OR suppress* OR ruminat*)

3 AB affect AND (regulat* or dysregulat* or generat* OR modulat* OR adapt* OR cop* OR react* OR arous* OR avoid* OR behavio* OR suppress* OR ruminat*)

4 AB "self-concept" OR "mood regulat*" OR "situation selection" OR "situation modification" OR "attentional deployment" OR "response modulation" OR "problem focused coping" OR "impulsivity" OR "hyper-reactivity" OR "anger regulation" OR "experiential avoidance" OR "expressive suppression" OR "thought suppression" OR "ruminat*" OR "emotion regulation questionnaire" OR "Difficulties in Emotion Regulation Scale" OR "emotion regulation strategy"

5 S2 or S3 or S4

6 AB treat* or interven* or "early intervention" or "self-directed intervention" or "self-directed" or "public health"

7 AB phon* or mobile* or "smart-phone*" or device* or computer* or tablet* or "device-based" or "app-based" or apps or app or "sensor*" or "social media"

8 AB (digital or virtual or online or interactive) and (health* or intervention or support or platform*)

9 AB "eHealth" or "e-health" or "m-health" or mHealth or "tele-health" or telehealth

10 S7 or S8 or S9

11 S1 and S5 and S6 and S10

Limiters - Published Date: 19950101-; English Language; Peer Reviewed; Research Article; Human; Publication Type: Journal Article; Age Groups: Child: 6-12 years, Adolescent: 13-18 years; Language: English

Database: The Cochrane Central Register of Controlled Trials (CENTRAL)

Search Strategy:

----

1 adolescen* or juvenil* or youth* or teen* or pubescen* or pediatric* or paediatric* or "high school" or "secondary school" or school* or "young people" or "young person*" or "young adult*" or "young man" or "young men" or "young woman" or "young women" or student*

2 emotion* AND (regulat* or dysregulat* or generat* OR modulat* OR adapt* OR cop* OR react* OR arous* OR avoid* OR behavio* OR suppress* OR ruminat*)

3 affect AND (regulat* or dysregulat* or generat* OR modulat* OR adapt* OR cop* OR react* OR arous* OR avoid* OR behavio* OR suppress* OR ruminat*)

4 self-concept OR "mood regulat*" OR "situation selection" OR "situation modification" OR "attentional deployment" OR "response modulation" OR "problem focused coping" OR "impulsivity" OR hyper-reactivity OR "anger regulation" OR "experiential avoidance" OR "expressive suppression" OR "thought suppression" OR ruminat* OR “emotion regulation questionnaire” OR “Difficulties in Emotion Regulation Scale” OR “emotion regulation strategy”

5 #2 or #3 or #4

6 treat* or interven* or "early intervention" or "self-directed intervention" or "self-directed" or "public health"

7 phon* or mobile* or "smart-phone*" or device* or computer* or tablet* or "device-based" or "app-based" or apps or app or "sensor*" or "social media"

8 (digital or virtual or online or interactive) and (health* or intervention or support or platform*)

9 "eHealth" or "e-health" or "m-health" or mHealth or "tele-health" or telehealth

10 #7 or #8 or #9

11 #1 and #5 and #6 and #10

with Publication Year from 1995 to 2023, in Trials with Public Health, Developmental, Psychosocial and Learning Problems, Child Health in Cochrane Groups

Database: Web of Science: Core Collection

Search Strategy:

----

1 AB=(adolescen* or juvenil* or youth* or teen* or pubescen* or pediatric* or paediatric* or "high school" or "secondary school" or school* or "young people" or "young person*" or "young adult*" or "young man" or "young men" or "young woman" or "young women" or student*)

2 AB=(emotion* AND (regulat* or dysregulat* or generat* OR modulat* OR adapt* OR cop* OR react* OR arous* OR avoid* OR behavio* OR suppress* OR ruminat*))

3 AB=(affect* AND (regulat* or dysregulat* or generat* OR modulat* OR adapt* OR cop* OR react* OR arous* OR avoid* OR behavio* OR suppress* OR ruminat*))

4 AB=(self-concept OR "mood regulat*" OR "situation selection" OR "situation modification" OR "attentional deployment" OR "response modulation" OR "problem focused coping" OR "impulsivity" OR hyper-reactivity OR "anger regulation" OR "experiential avoidance" OR "expressive suppression" OR "thought suppression" OR ruminat* OR “emotion regulation questionnaire” OR “Difficulties in Emotion Regulation Scale” OR “emotion regulation strategy”)

5 #2 OR #3 OR #4

6 AB=(treat* or interven* or "early intervention" or "self-directed intervention" or "self-directed" or "public health")

7 AB=(phon* or mobile* or "smart-phone*" or device* or computer* or tablet* or "device-based" or "app-based" or apps or app or "sensor*" or "social media")

8 AB=((digital or virtual or online or interactive) and (health* or intervention or support or platform*))

9 AB=("eHealth" or "e-health" or "m-health" or mHealth or "tele-health" or telehealth)

10 #9 OR #8 OR #7

11 #10 AND #6 AND #5 AND #1

18 #17 and 2023 or 2022 or 2021 or 2020 or 2019 or 2000 or 2001 or 2002 or 2003 or 2004 or 2005 or 2006 or 2007 or 2008 or 2009 or 2010 or 2011 or 2012 or 2013 or 2014 or 2015 or 2016 or 2017 or 2018 (Publication Years) and English (Languages) and English (Languages) and Article (Document Types)

19 #10 AND #6 AND #5 AND #1 and 2023 or 2022 or 2021 or 2020 or 2019 or 2018 or 2017 or 2016 or 2015 or 2014 or 2013 or 2012 or 2011 or 2001 or 2002 or 2003 or 2004 or 2006 or 2007 or 2009 or 2010 or 2005 or 2008 or 1995 or 1996 or 1997 or 1998 or 1999 or 2000 (Publication Years) and Psychiatry or Public Environmental Occupational Health or Education Educational Research or Psychology Multidisciplinary or Psychology Clinical or Pediatrics or Health Care Sciences Services or Psychology Developmental or Psychology or Family Studies or Multidisciplinary Sciences or Nursing or Medicine Research Experimental or Medicine General Internal or Psychology

Educational or Behavioral Sciences or Education Scientific Disciplines or Health Policy Services or Social Sciences Interdisciplinary or Social Work or Education Special or Psychology Social or Psychology Applied or Psychology Experimental or Sociology (Web of Science Categories) and Book Review or Data Paper or Reprint or Editorial Material or Book Chapters or Biographical-Item or Review Article or Proceeding Paper or Early Access (Exclude – Document Types) and English (Languages) and Psychology or Psychiatry or Public Environmental Occupational Health or Health Care Sciences Services or Education Educational Research or Pediatrics or General Internal Medicine or Nursing or Family Studies or Science Technology Other Topics or Research Experimental Medicine or Behavioral Sciences or Social Sciences Other Topics or Social Work (Research Areas)

Database: Elsevier Embase 1947 to present

Search Strategy:

----

1 (adolescen* or juvenil* or youth* or teen* or pubescen* or pediatric* or paediatric* or "high school" or "secondary school" or school* or "young people" or "young person*" or "young adult*" or "young man" or "young men" or "young woman" or "young women" or student*).ti,ab

2 (emotion* AND (regulat* or dysregulat* or generat* OR modulat* OR adapt* OR cop* OR react* OR arous* OR avoid* OR behavio* OR suppress* OR ruminat*)).,ti,ab

3 (affect AND (regulat* or dysregulat* or generat* OR modulat* OR adapt* OR cop* OR react* OR arous* OR avoid* OR behavio* OR suppress* OR ruminat*)).,ti,ab

4 ("self-concept" OR "mood regulat*" OR "situation selection" OR "situation modification" OR "attentional deployment" OR "response modulation" OR "problem focused coping" OR "impulsivity" OR "hyper-reactivity" OR "anger regulation" OR "experiential avoidance" OR "expressive suppression" OR "thought suppression" OR "ruminat*" OR "emotion regulation questionnaire" OR "Difficulties in Emotion Regulation Scale" OR "emotion regulation strategy").ti,ab

5 2 or 3 or 4

6 (treat* or interven* or "early intervention" or "self-directed intervention" or "self-directed" or "public health").ti,ab.

7 phon* or mobile* or "smart-phone*" or device* or computer* or tablet* or "device-based" or "app-based" or apps or app or "sensor*" or "social media").ti,ab.

8 ((digital or virtual or online or interactive) and (health* or intervention or support or platform*)).ti,ab.

9 ("eHealth" or "e-health" or "m-health" or mHealth or "tele-health" or telehealth).ti,ab.

10 7 or 8 or 9

11 1 and 5 and 6 and 10

12 limit 11 to (human and english language and "remove medline records" and embase and yr="1995 -Current" and journal)

Database: EThOS (PhD Theses)

Search Strategy:

1	"adolescen\" OR "high school" OR "secondary school" OR "youth" OR "young person" OR "young people"	Any word
2	AND "emotional regulation" OR "mood regulat\"	Any word
3	OR "emotion\" AND ("dysregulat\" OR "generat\" OR "modulat\" OR "adapt\" OR "cop\" OR react\ OR arous\ OR avoid\ OR behavio\ OR suppress\" OR "ruminat\")	Any word
4	OR affect AND (regulat\ or dysregulat\ or generat\ OR modulat\ OR adapt\ OR cop\ OR react\ OR arous\ OR avoid\ OR behavio\ OR "suppress\" OR "ruminat\")	Any word
5	AND "prevent\" OR "promot\" OR "treat\" OR "evaluat\" OR "effective\" OR "interven\" OR "early intervention" OR "public health" OR "therap\"	Any word
6	AND "telemedicine\" OR "e-health\" OR "app-based" OR "virtual\" OR "web-based" OR "online\" OR "wearable" OR "device" OR "digital" OR "remote"	Any word

Database: OATD (PhD Theses)

Search Strategy:

1	"adolescen*" OR "youth*" OR "high school" OR "secondary school" OR "young person" OR "young people"	Any word
2	AND ("emotional regulation" OR "mood regulat*") OR ("emotion*" AND ("dysregulat*" OR "generat*" OR "modulat*" OR "adapt*" OR "cop*" OR react* OR arous* OR avoid* OR behavio* OR suppress*" OR "ruminat*")) OR (affect AND (regulat* or dysregulat* or generat* OR modulat* OR adapt* OR cop* OR react* OR arous* OR avoid* OR behavio* OR "suppress*" OR "ruminat*"))	Any word
3	AND "prevent*" OR "promot*" OR "treat*" OR "evaluat*" OR "effective*" OR "interven*" OR "early intervention" OR "public health" OR "therap*"	Any word
4	AND "telemedicine/" OR "e-health/" OR "app-based" OR "virtual*" OR "web-based" OR "online*" OR "wearable" OR "device" OR "digital" OR "remote"	Any word

Database: HMIC Health Management Information Consortium *1979 to November 2022*

Search Strategy:

----

1 exp adolescents/ or adolescent development/ or young adults/ or youth/

2 (adolescen* or juvenil* or youth* or teen* or pubescen* or pediatric* or paediatric* or "high school" or "secondary school" or school* or "young people" or "young person*" or "young adult*" or "young man" or "young men" or "young woman" or "young women" or student*).ti,ab.

3 1 or 2

4 “emotional regulation”.mp.

5 (emotion* AND (regulat* or dysregulat* or generat* OR modulat* OR adapt* OR cop* OR react* OR arous* OR avoid* OR behavio* OR suppress* OR ruminat*)).ti,ab.

6 (affect AND (regulat* or dysregulat* or generat* OR modulat* OR adapt* OR cop* OR react* OR arous* OR avoid* OR behavio* OR suppress* OR ruminat*)).ti,ab

7 ("self-concept" OR "mood regulat*" OR "situation selection" OR "situation modification" OR "attentional deployment" OR "response modulation" OR "problem focused coping" OR impulsivity OR "hyper-reactivity" OR "anger regulation" OR "experiential avoidance" OR "behavio* avoidance" OR "expressive suppression" OR "thought suppression" OR ruminat* OR “emotion regulation questionnaire” OR “Difficulties in Emotion Regulation Scale” OR “emotion regulation strategy”).ti,ab.

8 4 or 5 or 6 or 7

9 (prevent* or promot* or treat* or evaluat* or effective*).ti,ab.

10 (interven* or "early intervention" or "public health" or therap*).ti,ab.

11 9 or 10

12 telemedicine/ or e-health/

13 (phon* or mobile* or "smart-phone*" or device* or computer* or tablet* or "device-based" or "app-based" or apps or app or "sensor*" or "smart-home*" or "social media").ti,ab.

14 (tele and (phone* or nursing or health* or medicine or intervention or informatics or care or support or monitor* or presence or consultat* or rehab* or communicat*)).ti,ab.

15 (digital and (nursing* or health* or medicine or intervention or informatics or care or support or monitor* or presence or consultat* or rehab* or communicat* or platform*)).ti,ab.

16 (virtual and (nursing* or health* or medicine or intervention or informatics or care or support or monitor* or presence or consultat* or rehab* or communicat* or reality* or platform*)).ti,ab.

17 (online and (nursing* or health* or medicine or intervention or informatics or care or support or monitor* or presence or consultat* or rehab* or communicat* or platform*)).ti,ab

18 (interactiv* or "eHealth" or "e-health" or "e-therapy" or "e-learn" or "m-health").ti,ab.

19 ("wireless health" or "health telematics" or "healthcare technology" or "therapeutic technology" or online or "medical informatics" or "medical information system" or "electronic health information" or "mobile application*" or "web-based" or website or wifi or "internet-delivered" or "internet-based" or "smart toy" OR “wearable*”).ti,ab.

20 12 or 13 or 14 or 15 or 16 or 17 or 18 or 19

21 3 and 8 and 11 and 20

22 limit 21 to (English language and yr=”2000-2022”)

Database: PsyArXiv

Search Strategy:

("emotional regulation" OR "mood regulat*" OR "emotion dysregulat*" OR "arous*" OR "avoid*" OR "behavio*" OR "suppress*" OR "ruminat*") AND ("prevent*" OR "promot*" OR "treat*" OR "evaluat*" OR "effective*" OR "interven*" OR "early intervention" OR "public health" OR "therap*") AND ("telemedicine" OR "e-health" OR "app-based" OR "virtual*" OR "web-based" OR "online*" OR "wearable" OR "device" OR "digital" OR "remote")

Database: TRIP

Search Strategy:

("adolescen*" OR "youth*" OR "high school" OR "secondary school" OR "young person" OR "young people") AND ("emotional regulation" OR "mood regulat*" OR "emotion dysregulat*" OR "arous*" OR "avoid*" OR "behavio*" OR "suppress*" OR "ruminat*") AND ("prevent*" OR "promot*" OR "treat*" OR "evaluat*" OR "effective*" OR "interven*" OR "early intervention" OR "public health" OR "therap*") AND ("telemedicine" OR "e-health" OR "app-based" OR "virtual*" OR "web-based" OR "online*" OR "wearable" OR "device" OR "digital" OR "remote")

Database: ClinicalTrials.gov

Search Strategy:

("telemedicine" OR "e-health" OR "virtual*" OR "digital") | Emotional Regulation | Child

Database: ACM Digital Library

Search Strategy:

[[All: "emotional regulation"] OR [All: "mood regulat*"] OR [All: "emotion dysregulat*"]] AND [[All: "prevent*"] OR [All: "promot*"] OR [All: "treat*"] OR [All: "evaluat*"] OR [All: "effective*"] OR [All: "interven*"] OR [All: "early intervention"] OR [All: "public health"] OR [All: "therap*"]] AND [[All: "adolescen*"] OR [All: "youth*"] OR [All: "high school"] OR [All: "secondary school"] OR [All: "young person"] OR [All: "young people"]] AND [[All: "app-based"] OR [All: "virtual*"] OR [All: "web-based"] OR [All: "online*"] OR [All: "digital"] OR [All: "remote"]]
